# Effects of ATP7A overexpression in mice on copper transport and metabolism in lactation and gestation

**DOI:** 10.1002/phy2.195

**Published:** 2014-01-13

**Authors:** Jarrod Wadwa, Yu‐Hsiang Chu, Nhu Nguyen, Thomas Henson, Alyssa Figueroa, Roxana Llanos, Margaret Leigh Ackland, Agnes Michalczyk, Hendrik Fullriede, Grant Brennan, Julian F. B. Mercer, Maria C. Linder

**Affiliations:** 1 Department of Chemistry and Biochemistry, California State University Fullerton, Fullerton, 92834‐6866, California; 2 Centre for Cellular and Molecular Biology, Deakin University, Burwood, 3125, Victoria, Australia

**Keywords:** Ceruloplasmin metalation, copper transport, ferroxidases, Menkes protein, milk

## Abstract

Placentae and mammary epithelial cells are unusual in robustly expressing two copper “pumps”, ATP7A and B, raising the question of their individual roles in these tissues in pregnancy and lactation. Confocal microscopic evidence locates ATP7A to the fetal side of syncytiotrophoblasts, suggesting a role in pumping Cu towards the fetus; and to the basolateral (blood) side of lactating mammary epithelial cells, suggesting a role in recycling Cu to the blood. We tested these concepts in wild‐type C57BL6 mice and their transgenic counterparts that expressed hATP7A at levels 10–20× those of endogenous mAtp7a. In lactation, overexpression of ATP7A reduced the Cu concentrations of the mammary gland and milk ~50%. Rates of transfer of tracer ^64^Cu to the suckling pups were similarly reduced over 30–48 h, as was the total Cu in 10‐day ‐old pups. During the early and middle periods of gestation, the transgenic litters had higher Cu concentrations than the wild‐type, placental Cu showing the reverse trend; but this difference was lost by the first postnatal day. The transgenic mice expressed ATP7A in some hepatocytes, so we investigated the possibility that metalation of ceruloplasmin (Cp) might be enhanced. Rates of ^64^Cu incorporation into Cp, oxidase activity, and ratios of holo to apoceruloplasmin were unchanged. We conclude that in the lactating mammary gland, the role of ATP7A is to return Cu to the blood, while in the placenta it mediates Cu delivery to the fetus and is the rate‐limiting step for fetal Cu nutrition during most of gestation in mice.

## Abstract (precis)

Copper transport to the fetus and newborn via the placenta and milk is critical for life and development. Cells in placenta and mammary epithelium are somewhat unique in expressing both ATP7A and ATP7B, which participate in copper transport. Using transgenic mice overexpressing ATP7A, our studies show that in placenta, ATP7A is rate‐limiting for copper transport to the fetus, while in the mammary epithelium it returns excess copper to the blood.

## Introduction

The copper “pumps”, ATP7A and ATP7B, are vital for specific processes in mammals. Their dysfunction results in well‐known genetic diseases of copper deficiency and overload, respectively: Menkes disease (MD) occurs when ATP7A is mutated, and Wilson disease (WD) when ATP7B is not functioning (La Fontaine and Mercer 2007; Gupta and Lutsenko 2009; LaFontaine et al. 2010. Both P‐type ATPases efflux copper from cells by pumping it from the cytosol and/or via exocytosis. They also supply copper to cupro‐enzymes in the secretory pathway. Menkes disease and the less deadly Occipital Horn Syndrome (OHS) are the result of a severe copper deficiency because of a marked decrease in the ability of copper to exit intestinal enterocytes to enter the blood, after absorption from the diet, compounded by maldistribution of copper to internal organs. Boys with MD usually die within 3 years of birth due to severe impairment of physiological functions dependent on copper enzymes. In contrast, the primary consequence of WD is excessive accumulation of copper in the liver and some other organs. This accumulation is due to impairment of copper excretion, which occurs primarily through the bile (Roelofsen et al. 2000; La Fontaine and Mercer 2007; Gupta and Lutsenko 2009; LaFontaine et al. 2010; Linder 2010). Copper accumulation leads to oxidative stress and toxicosis. Untreated humans typically die from liver failure in their teens, although a neurological form of WD results from accumulation of copper in the brain.

In mammals, ATP7A is expressed in most cells, while ATP7B has a much more restricted expression (La Fontaine and Mercer 2007; Lutsenko et al. 2007; Gupta and Lutsenko 2009; LaFontaine et al. 2010). Both are found in membranes of the trans‐Golgi network (TGN) as well as in vesicles cycling to and from portions of the plasma membrane. At the TGN, both proteins supply copper to secreted cupro‐enzymes such as lysyl oxidase (ATP7A) and ceruloplasmin (ATP7B). If copper levels rise in the cell, both proteins move onto vesicles, which traffic to the plasma membrane to release excess copper. This process, termed copper‐induced trafficking, is central to the maintenance of copper homeostasis (La Fontaine and Mercer 2007). In the intestine, copper absorbed by enterocytes from the diet is carried by the cytosolic copper chaperone ATOX1 to ATP7A in the TGN, and from there it enters endosomes destined to empty copper into the blood via exocytosis (Lutsenko et al. 2007; Nyasae et al. 2007; Gupta and Lutsenko 2009; LaFontaine et al. 2010; Lutsenko 2010). The form of copper released has not been characterized, but is capable of direct binding to the blood plasma proteins, albumin and transcuprein that transport it (Linder 2010), suggestive of Cu(II) ions. In hepatocytes, copper is chaperoned (also by ATOX1) to ATP7B in the TGN and/or endosomes, from where it is exocytosed to that portion of the plasma membrane constituting the bile canaliculi (La Fontaine and Mercer 2007; Lutsenko et al. 2007; Gupta and Lutsenko 2009; LaFontaine et al. 2010; Linder 2010). ATPB, however, is also essential for metalation of ceruloplasmin (Cp) produced by hepatocytes and secreted into the blood plasma (Yamada et al. 1993; Terada et al. 1998). Lysyl oxidase produced by fibroblasts for modification of collagen and elastin in the extracellular matrix receives copper from ATP7A; hence patients with MD and OHS have a pronounced connective tissue weakness (Kaler 2011; Kodama et al. 2012).

While most mammalian cells express either ATP7A or B, a few have robust expression of both. These include the mammary epithelial cell that produces milk (Hardman et al. 2007) and the placental syncytiotrophoblasts that transfer nutrients from the blood of the mother to the fetus (Hardman et al. 2004). Prior studies from our laboratories have shown that in the (lactating) human breast (Ackland et al. 1999) as well as in transgenic (Tg) mice with 10–20× more human ATP7A than murine Atp7a (Llanos et al. 2008), ATP7A localized to the maternal blood side of the mammary epithelium, merging with integrin in confocal microscopy. In contrast, ATP7B was found mainly in the TGN and in endosomes located closer to the apical side, where milk is released. This localization suggested that during lactation, ATP7B is mainly concerned with providing copper to milk secretions. The copper released into milk would in part be associated with Cp, which is not just in blood but is also a major milk copper protein made by the mammary epithelium (Wooten et al. 1996; Cerveza et al. 2000). As in hepatocytes, apoceruloplasmin is likely to be metalated in the TGN of mammary cells with copper provided by ATP7B, followed by exocytosis, as toxic milk mice (without Atp7b) have much less copper in their milk (Rauch 1983; Michalczyk et al. 2000). Other forms of copper destined for milk might undergo a similar process, although a direct pumping of copper ions by ATP7B across the apical (brush border) membrane into the developing milk cannot be excluded. (The copper‐binding components of milk have not yet been adequately defined.) Localization of ATP7A primarily on the other side of the cell (with or without lactation) might indicate it is effluxing copper from the mammary epithelium back into the blood.

Our prior studies on syncytiotrophoblasts of third trimester human placenta from our laboratories (Hardman et al. 2006, 2011) indicated that ATP7A (and CTR1) localizes to the fetal side of the human placenta (with Na+/K+ATPase) and not to the maternal side (with transferrin receptor). In contrast, ATP7B was found by transmission electron microscopy to be primarily in microvilli of the syncytiotrophoblast, with a small proportion within the cytoplasm adjacent to the nuclei (Hardman et al. 2011). This suggested that ATP7A might be transporting copper from the placenta to the fetal circulation, while ATP7B might be metalating a unique form of Cp expressed in placenta (Yang et al. 1990) as well as its membrane‐bound homologs, hephaestin and zyklopen, the latter also placenta‐specific (Anderson et al. 2002; Chen et al. 2004, 2010). In addition, it might play a role in returning copper to the maternal circulation.

The study reported here was designed to test two hypotheses about ATP7A. First that in the placenta, ATP7A mediates the flow of copper from the mother to the fetus by pumping or exocytosing copper in the direction of the fetal blood circulation, and that this process could be rate‐limiting for the copper nutrition of the fetus. Second that in the mammary epithelium, ATP7A returns copper to the blood, to mitigate against accumulating copper in mammary epithelium – for example, when lactation ceases, and prevents an excess of copper from entering the milk. To test these hypotheses, we utilized the Tg mice that produced 10–20× higher amounts of human ATP7A than the endogenous mAtp7a (Llanos et al. 2008) and compared the results side by side with those for the wild type (WT). The results support our hypotheses about the functions of ATP7A in mammary epithelium and placenta, and suggest that ATP7A may be rate‐limiting for copper transport to the fetus.

## Methods

### Animal and treatments

Experiments were performed on WT and Tg mice expressing hATP7A produced in the C57BL/6J background strain, as previously described and characterized (Llanos et al. 2008). In these mice, levels of hATP7A were shown to be 10–20× higher than endogenous levels of Atp7A by Western blotting. Pregnant, lactating, and virgin female mice ranged from 2.5 to 8 months of age. All studies were preapproved by the university IACUC. To study copper transport, trace amounts (<1 ng) of radioactive ^64^Cu(II) (20–100 *μ*Ci) were administered i.p. as the nitrilotriacetate (NTA) complex (5–10:1 NTA:Cu molar ratio), and mice were euthanized at various times thereafter. In the case of lactating mice, milk was obtained under ketamine/xylazene or pentobarbital anesthesia, after i.p. injection of 1U oxytocin, within the last 15 min before euthanasia by exsanguination. Plasma was obtained from heparinized blood collected from the vena cava. Major organs (liver, kidney, spleen, lung, brain, skeletal muscle, mammary gland, and in some cases also skin and skeleton) were collected for counting distribution of radioactive copper tracer. Percent dose of tracer ^64^Cu in a given organ or pup/fetus was calculated based on the total radioactivity recorded in all major organs of the dam, plus offspring. Whole pups were collected during days 1–10 of lactation. In the case of pregnant mice, half the uterus (with its placentae and pups) was collected as a whole, making note of the number of pups; the other half was dissected to separately collect fetuses and placentae. Samples were stored frozen at −20^o^C. Radioactivity was measured in a gamma counter (Perkin Elmer, Model Cobra II, St. Louis, MO).

### Rates of ceruloplasmin synthesis

Incorporation of ^64^Cu into plasma Cp was measured 2 h after i.p. injection of isotope. The resulting Cp was isolated from plasma after first removing endogenous immunoglobulins with protein A beads, then treating with anti‐human Cp antibody (Sigma‐Aldrich, Waltham, MA) and precipitating with protein A beads. Washed precipitates were counted for radioactivity. Alternatively, equal volumes of plasma were applied to nondenaturing tube gel electrophoresis, gels sliced, and the radioactivity in the Cp band (Rf 0.6) was counted (Middleton and Linder 1993). In both cases, the results were used to calculate ^64^Cu incorporated into the Cp of 1 mL plasma over 2 h. Data from combined experiments are given as percent of the mean for WT (control) mice in a given experiment.

### Copper analysis

Concentrations of copper in samples were determined by graphite furnace atomic absorption spectrometry, as previous described (Chu et al. 2011). Homogenates of tissues and whole fetuses or pups were wet ashed with trace element‐grade nitric acid, prior to quantitation of copper in the 1–2% nitric acid‐dissolved mineral residue.

### Detection of apo and holoCp

Nontraditional sodium dodecyl sulphate polyacrylamide gel electrophoresis (SDS‐PAGE, without boiling samples) was performed to separate apo and holoCp based on previous work by our own and other laboratories (Sato and Gitlin 1991; Middleton and Linder 1993; Hirano et al. 2005). Nontraditional and traditional SDS‐PAGE (with boiling) was carried out in 7.5% acrylamide resolving gels and transferred to PVDF membranes, followed by immunoblotting with primary rabbit anti‐mouse Cp antibody (Biomatik; Wilmington, DE) and goat anti‐rabbit‐horse radish peroxidase conjugate (Bio‐Rad, Hercules, CA), developing with peroxide and Luminal Enhancer solution from Thermo Scientific (Rockford, IL). Images were captured by Kodak Image Station 4000R (Molecular Bioimaging, Upland, CA).

### Cp enzyme activity assays

p‐Phenylene‐diamine (pPD) oxidase and ferroxidase activities of whole plasma were measured as previously described (Gray et al. 2009), the latter using an adaptation of the Erel assay (Erel 1998). In the pPD assay, oxidation of substrate, at pH 5.5 and 37°C, was followed at 540 nm. Ferroxidation of Fe(II) (70 *μ*mol/L ferrous ammonium sulfate) at pH 5.96 and 37°C was followed by measuring the Fe(II) remaining from 1 to 5 min after the start of substrate addition, stopping the reaction with Ferene S, and reading absorbance of the complex at 600 nm. In some cases, Na azide (1 mmol/L) was added to the reaction.

### Statistics

Data are presented as Mean ± SD for the indicated number of determinations/samples. Statistical significance was determined by analysis of variance (ANOVA) and Student's *t* Test, *P* < 0.05 being considered significant.

## Results

### Effects of excess ATP7A on copper transport in lactation

#### Effects on the copper contents of mammary gland and milk

**Figure 1.**
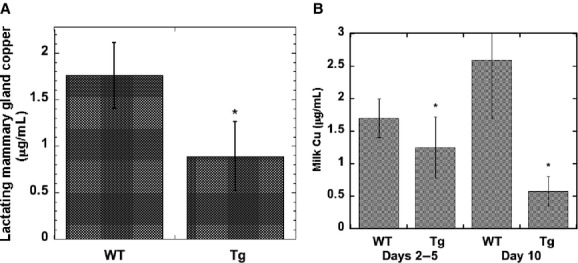
Effects of ATP7A overexpression on copper concentrations of lactating mammary gland and milk. (A) Copper concentrations of lactating mammary gland (2–5 days post partum) from wild‐type (WT) and transgenic (Tg) mice, in *μ*g/g. Values are Mean ± SD (*N* = 16). *Statistically significant decrease (*P* < 0.005). (B) Copper concentrations of milk (*μ*g/mL) early (days 2–5) and later (day 10) in lactation of WT and Tg mice. Mean ± SD, for 10 WT, 9 Tg mice (Days 2–5), and 3 WT and 3 Tg mice (Day 10). *P < 0.003 for differences between WT and Tg milk

We had postulated that ATP7A would enhance efflux of copper back into the blood; so we expected levels of total copper to be lower in the Tg mice, which is what we found. Similar to our previously published report for day 7–8 of lactation (Llanos et al. 2008) (days 7–8 after birth), overexpression of ATP7A lowered copper concentrations about 50% (*P* < 0.005), in this case in the mammary gland of mice analyzed on days 2–5 of lactation (Fig. 1A). We also expected that less copper in the mammary epithelial cells would result in less transfer of copper into milk. The results of analyzing many samples of milk taken on days 2–5 as well as 10 days after birth are consistent with what we expected and shown in Figure 1B. Concentrations of copper in milk were 25–70% lower in samples from mice with extra ATP7A.

#### Effect of excess ATP7A on transfer of tracer copper from mother to newborn during suckling

**Figure 2.**
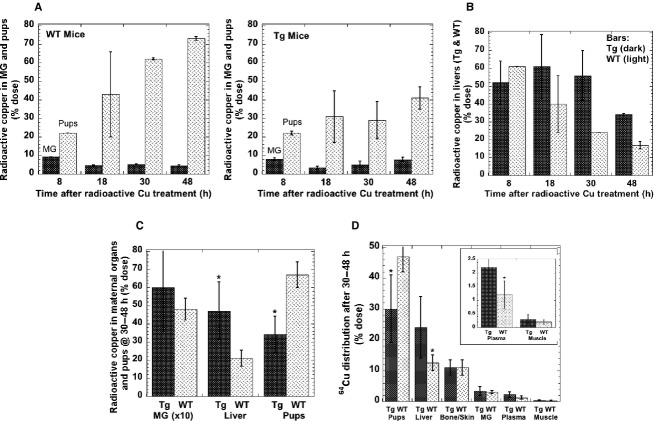
Effect of ATP7A overexpression transport of copper to liver, mammary gland and pups in lactation (days 2–5), using ^64^Cu. (A) ^64^Cu in maternal mammary gland (MG; dark bars) and suckling pups (light bars) at different times after i.p. injection of the dam. Data are percent of ^64^Cu dose (Means ± AD or SD, for 2–4 litters per time point). (B) Copper accumulation in livers of Tg (dark bars) and WT (light bars) mice at various times after tracer ^64^Cu injection (Mean ± AD or SD, for 2–4 litters per time point. (C) Cumulative data for the 30 and 48 h time points for mammary gland (MG) ×10, livers and pups of Tg and WT mice. Data are percent of ^64^Cu dose administered (Means ± SD, for 6 litters; 2 time points) *P < 0.001 for difference between WT and Tg mice. (D) Transfer of ^64^Cu tracer to maternal organs and pups when litters were culled to 5 pups at time of injection, for Tg (dark bars) and WT (light bars) mice. Combined data for 30 and 48 h after injection**.** Percent dose in litters. Mean ± SD for four WT and seven Tg litters *Significant differences between WT and Tg: from left to right: *P* < 0.02, *P* < 0.02, *P* < 0.04

The effects of excess ATP7A on copper transfer into milk and to offspring was also examined using trace amounts of radioactive copper (^64^Cu) injected into the dams. Uptake of tracer by the mammary gland and liver, and its accumulation in the suckling litters was monitored over 48 h. Figure 2A shows that in both the WT and Tg mice (overexpressing ATP7A), the mammary gland (dark bars) accumulated between 5% and 10% of the administered dose of ^64^Cu at any of the times examined. In contrast, accumulation of radiotracer in the suckling pups (light bars) increased over time. The rate of increase was, however, much more rapid in the case of the WT, reaching about 70% of the total initial dose of tracer administered, by 48 h. This was almost twice as much as the accumulation of tracer in the litters of the Tg mice. Conversely, as seen in Figure 2B, the livers of the Tg mice (dark bars) retained a larger proportion of the radioactive copper than the WT at later times. It is noteworthy that the differences in ^64^Cu contents in liver and pups of the WT and Tg mice was not evident at the earlier, that is, it developed over time. At 8 h after injection about 20% of the total dose accounted for in the major organs of the dam plus pups had been transferred to the litters (Fig. 2A), and this was true for both the WT and transgenics. However, over the next 40 h, the total percentage of tracer ^64^Cu in the WT pups increased 3.5×, while that entering the Tg litter only doubled. A summary of the data for differences between ^64^Cu ending up in liver, pups, and mammary gland after 30 and 48 h is given in Figure 2C. For liver and pups but not mammary gland, the differences were highly significant; and it is noteworthy that the drop in total transfer of radiocopper to the pups matched the percent dose of radiocopper retained by/returned to the maternal liver.

As in these studies the size of the litters varied from four to seven, we carried out an additional experiment in which transfer of ^64^Cu from mother to suckling pups was measured with litters of identical size (culled to five pups per dam at the time of ^64^Cu injection of the dam). The same kinds of results were obtained (Fig. 2D). In this case, we also measured the percentages of radioactive copper tracer that became associated with the skeleton and skin of the dam. We are not aware that uptake of radioactive copper into these copper‐dependent tissues has ever been measured before, and we postulated they might represent a significant “sink” for copper. The data show that in both the lactating WT and Tg mice, a significant but not enormous portion (~10%) of the injected tracer ^64^Cu ended up in those parts of the organism 30–48 h after administration.

**Figure 3.**
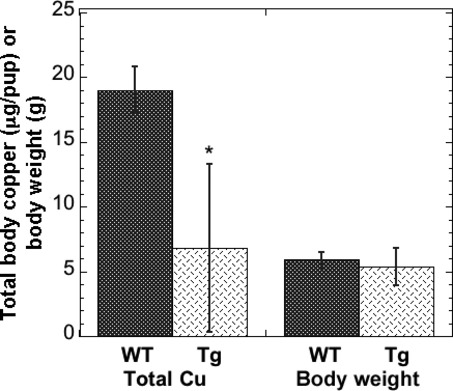
Effect of overexpression of ATP7A also leads to less total copper in the pups. Data for pups after 10 days of lactation. For the Tg mice, total Cu per pup (*μ*g) was significantly lower than that of the WT (*P* < 0.025), but there was no significant difference in body weight. Mean ± SD, *N* = 13 and 5, for WT and Tg litters, respectively.

As less radioactive copper tracer was being transferred from the dam to her litter when the mammary gland and other organs were expressing very high levels of ATP7A, we expected that the total copper levels in the pups would also be less than in the WT pups. This was confirmed by comparing the total copper contents of pups 10 days after birth (Fig. 3). Although there was no statistically significant difference in the weights of pups from the WT and Tg litter (Fig. 3, right), the total copper content of the pups was markedly and significantly less (Fig. 3, left), though also highly variable.

### Effects of excess ATP7A on copper transport during gestation

#### Total copper content of litters at various stages of gestation

**Figure 4.**
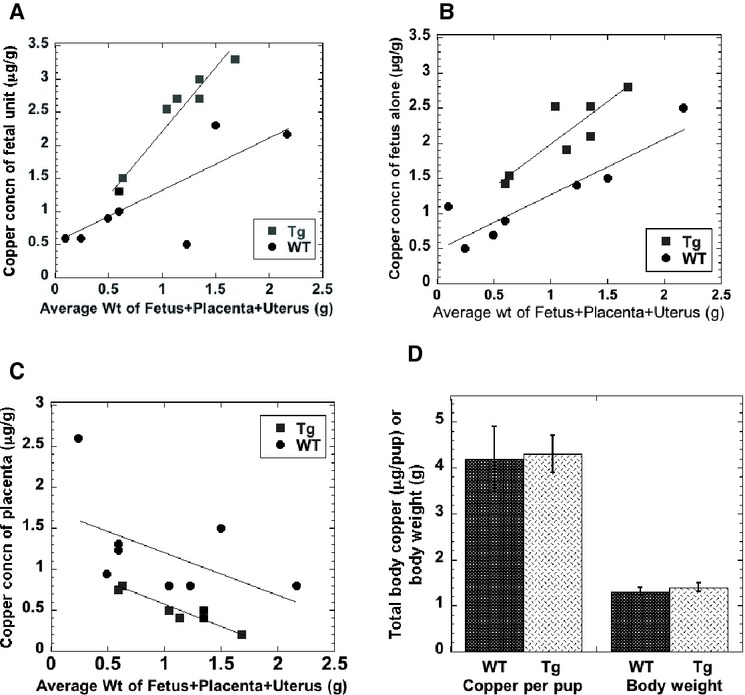
Effect of ATP7A overexpression on Cu in fetuses and placenta during gestation and at birth. (A and B) Concentrations of copper (*μ*g/g) in fetal units (A) and fetuses (B) from early to mid‐late gestation, in WT (crosses) and Tg (squares) mice. Gestational stage was indicated (on the *x*‐axis) in terms of the average weight (g) of a fetus plus its placenta and its portion of the uterus (a “fetal unit”) for a given litter. (C) Average copper concentrations of placentae for the same fetal units. (D) Total copper (*μ*g) and body weights (g) of newborn Tg (dark bars) and WT (light bars) pups: Mean ± SD, *N* = 13 and 18, respectively.

In a first attempt to explore whether, as hypothesized, ATP7A might normally be rate‐limiting for transfer of copper from mother to fetus, the copper contents of the litters of WT and Tg mice in utero were measured at various stages of pregnancy. As in early pregnancy it is very difficult to separate the fetuses from the placenta and uterus, the pups were collected in two different ways. For a given mouse, half the uterus (with a given number of pups) was analyzed as a whole; for the other half, fetuses, uterus, and placentae were separated out and separately analyzed. In Figure 4, the data obtained were plotted (*x*‐axis) as a function of the average weight of the fetus plus placenta plus uterine segment involved (fetal unit). This covered a large range of fetal weight and gestational stage, from very early to late in pregnancy. Figure 4A and B show that as we had predicted, copper concentrations of the fetal units in the Tg mice were higher at least during a major portion of the pregnancy. Moreover, the placentae from the ATP7A transgenic animals had less copper (Fig. 4C). These findings are consistent with the concept that increased expression of ATP7A caused an increase in the flow of copper to the fetuses from the placenta after its uptake from the blood of the dam, and that ATP7A is rate‐limiting for copper transfer to the fetus in pregnancy.

The concentrations of copper in the mammary gland of the Tg mice during gestation were also examined, and no particular trends were evident. However, overall, and similar to what we observed in lactation, the average concentrations of copper of the transgenic mammary gland during gestation was less than half that of the WT: 1.6 ± 1.3 versus 5.8 ± 3.0 *μ*g/g tissue (Mean ± SD, *N* = 8–10; *P* < 0.005).

If the rate of copper transfer to the fetus in gestation were enhanced by the excess ATP7A, we would expect that right after birth, the pups of the Tg dams would also have more copper. However, that did not prove to be the case. Figure 4D shows that on the first day after birth, the total body content of copper of the newborns was about the same for the WT and Tg mice. As already indicated, the data on fetal copper concentrations during gestation were plotted not against fetal body weight on the *x*‐axis, but against the weight of individual fetal units (a fetus with placenta and its portion of the uterus). If we take into consideration the actual fetal weights at the end of the time frame of gestation in which tissue was collected, this can give us an idea of the fetal growth rate before birth. When weights of the units were in the range of 1.7 to 2.2 g (the last prebirth units sampled), fetal weights averaged 1.2 g. One day after birth weights were in the range of 1.4 g. Thus, the average pup stood to gain about 15% of body weight from the end of gestation through first day of life. During this period, the WT pups seemed to make up the difference in total body copper content (compared to the Tg pups). Overall, the results imply that during most of gestation, ATP7A may be the rate‐limiting step in transfer of copper from mother to fetus.

### Effects of ATP7A overexpression on metalation and activity of plasma ceruloplasmin

In these Tg mice, some hepatocytes express ATP7A (Ke et al. 2006), which is not normally the case. As ATP7A like ATP7B pumps copper received from ATOX1 into the TGN where apoceruloplasmin is thought to receive its copper, it seemed possible that metalation of Cp might be enhanced by the ectopic expression of ATP7A in hepatocytes. Alternatively, expression of ATP7A in mouse hepatocytes might have the opposite effect, namely enhancing efflux of copper from hepatocytes, and thus reducing its availability to Cp. To test these possibilities, we examined several aspects of Cp metalation and metabolism.

**Figure 5.**
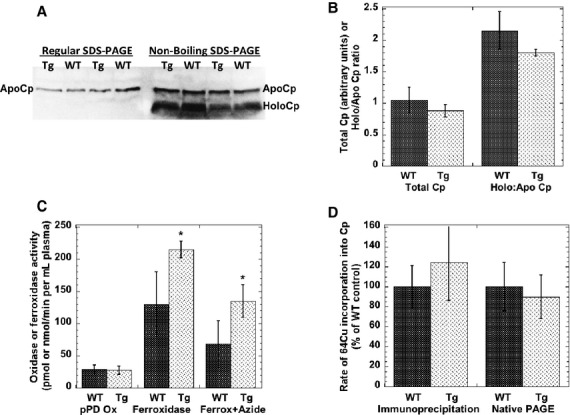
Expression, activity, Cu‐saturation of, and rate of Cu incorporation into ceruloplasmin (Cp), in Tg and WT mice. (A) Levels and copper saturation of Cp determined by Western blotting for Cp following SDS‐PAGE of equal volumes of denatured (left) or undenatured (not boiled; right) samples of plasma from WT and Tg mice (representative blots). Total levels of plasma Cp protein (132 kDa; left) and relative proportions of apo and holoCp in WT and Tg plasma are indicated. (*B*) Ratios of holo to apoCp calculated from densitometry of Western blots like those in (A). Mean ± SD (*N* = 4). *C*. Plasma p‐phenylene diamine (pPD) oxidase activity (pmol/min/mL) and ferroxidase activity (nmol/min/mL). Values are Mean ± SD, for *N* = 7–11, and *N* = 7–9, respectively. Ferroxidase activity was also measured in the presence of azide (*N* = 3 and 4 for WT and Tg mice, respectively). Azide significantly inhibited activity for plasma of both WT and Tg mice (*P* < 0.03). *D*. Rate of incorporation of ^64^Cu into plasma Cp over 2 h in Tg and WT mice, as determined by immunoprecipitation (left) or extraction of purified Cp from gels after native PAGE. Values are Mean ± SD (*N* = 5) as percent of the WT controls in a given experiment (*P* > 0.05).

Figure 5A (right side) shows a representative immunoblot depicting the relative ratios of apo to holoCp in the WT and Tg mice. Figure 5B shows the results of densitometry. There was no obvious difference, indicating no increased metalation of the Cp present. Total amounts of Cp protein per unit of plasma also did not differ (Fig. 5A, left side, and 5B). The oxidase activity of Cp, considered a good measure of the concentration of copper‐loaded Cp, was also compared (Fig. 5C, left) and no difference was observed between plasma from the Tg and WT mice. Rates of incorporation of tracer ^64^Cu into plasma Cp were also measured by two different procedures. Rates of copper incorporation into Cp for the WT and Tg mice, determined by two different methods, were indistinguishable (Fig. 5D). All of this indicates that at least at these levels of ATP7A expression in the Tg mouse hepatocytes, there was no change in metalation of Cp. Cp is also considered the major ferroxidase in blood plasma, although knocking out its gene only cuts total ferroxidase activity in half (Gray et al. 2009). In the ATP7A Tg mice, total ferroxidase activity appeared to be somewhat increased (Fig. 5C, right); but the increased ferroxidase in the Tg mice was not inhibited by azide (which inhibits Cp oxidase activity). As there was no increase in pPD oxidase activity, the increase in ferroxidase activity in the Tg mice is attributable to a ferroxidase that (unlike Cp) is resistant to high concentrations of azide. This suggests that expression of excess ATP7A may have resulted in increased copper metalation of a non‐Cp ferroxidase in the blood plasma.

**Figure 6.**
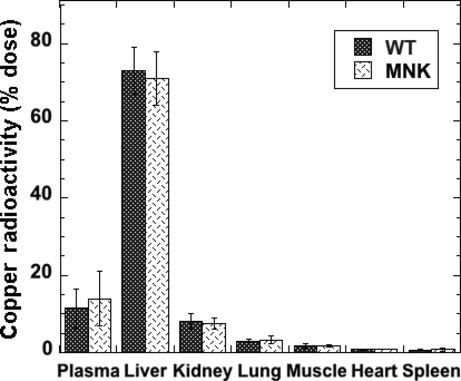
Excess ATP7A does not cause differences in rates of ^64^Cu tracer accumulation in body organs over 2 h. Radioactive copper in the major organs of WT (dark bars) and Tg (light bars) mice 2 h after ^64^Cu tracer injection (as percent of dose in the indicated organs and blood). Mean ± SD (*N* = 13 and 14). There were no statistically significant differences.

The presence of excess ATP7A transgene in nonpregnant and nonlactating mice did not alter the accumulation of tracer ^64^Cu in liver, kidney, and other organs (Fig. 6), in this case 2 h after injection. As has been demonstrated before for rats and mice (and humans) (Weiss and Linder 1985; Cabrera et al. 2008; Linder 2010), almost all the tracer initially entered the liver.

## Discussion

ATP7A is more widely expressed than ATP7B in mammalian cells. Earlier studies in whole animal and cultured cell models, as well as examination of human samples, indicated that the major role of ATP7A might be to transfer copper out of cells into the blood. Thus, for example, lack of active ATP7A, as in MD and Atp7a‐mutant mice (La Fontaine and Mercer 2007; Lutsenko et al. 2007; Kaler 2011), results in a profound decrease in transfer of dietary copper from intestinal absorptive cells into the blood, and a severe or fatal copper deficiency. In this condition, cells in most organs suffer from a lack of copper due to lack of absorption, though some accumulate copper due to lack of efflux – such as those of the kidney proximal tubules and blood–brain barrier.

After copper is released into the blood mediated by ATP7A across membranes, it associates primarily with two plasma proteins, albumin and transcuprein (Linder et al. 1998; Liu et al. 2007; Linder 2010), which (in most mammals) have a high affinity for copper. The other copper “pump”, ATP7B, is normally expressed robustly in only a few cell types like hepatocytes. It too is linked to efflux of cellular copper, efflux from hepatocytes into the bile being a major process for regulating the overall copper status of the body (Roelofsen et al. 2000; La Fontaine and Mercer 2007; Lutsenko et al. 2007; Gupta and Lutsenko 2009; Linder 2010). ATP7B also provides copper to Cp, the most abundant copper‐containing protein in the blood formed by hepatocytes. Lack of active ATP7B in humans or in animal models, as well as in severe copper deficiency, markedly reduces the secretion of copper‐containing Cp from liver hepatocytes into the blood. Mammary epithelial cells express Cp (Jaeger et al. 1991; Cerveza et al. 2000), which enters the milk (Wooten et al. 1996). Thus, knocking out Cp results in a marked drop in milk copper concentrations (Chu et al. 2011), again suggesting that ATP7B is responsible for metalation of a secreted copper protein. Apart from copper in Cp, it is not known in what form copper is secreted into milk or excreted into bile, including whether it is only bound to proteins (Linder 2010). The same is the case for copper released from cells via ATP7A.

In the studies reported here, we tested the hypothesis that ATP7A is a copper efflux protein that even in the lactating mammary epithelium (during overall transfer of copper to the milk) pumps copper back into the blood. We also tested the hypothesis that ATP7A might be rate‐limiting for transfer of copper from placental cells to the fetus (across the basolateral membrane into the fetal circulation) during gestation, when substantial quantities of this trace element are needed in support of fetal growth and prenatal storage in liver (Linder 2010). The results of our studies on lactation provide strong support for our hypothesis that ATP7A effluxes copper from mammary epithelial cells back into the blood. When the hATP7A transgene was expressed in mice, copper concentrations of mammary gland and milk were reduced by about half; rates of transfer of tracer copper from dam to pups were also markedly reduced; and suckling pups from the Tg mothers accumulated much less copper than those of the WT during the first 10 days of lactation (although there was no differences in growth rate, as evident from body weight). Interestingly, when monitoring the rate of accumulation of radioactive copper tracer in the suckling pups, differences between the results for the WT and Tg litters only became significant at later times. Eight hours after injection, the same percentage of tracer was recovered in the pups of the WT and Tg litters, whereas in the next 22–40 h, the percentage increased about 3.5× in the WT, but only 1.5–2× in the litters of the Tg dams. This suggests that two different pools of ^64^Cu in the mammary epithelial cells may be entering milk at different rates, an initial (faster) one not affected by ATP7A, and a second one depleted by excess ATP7A pumping copper back into the blood. It is possible that the copper is entering these pools from two different blood plasma sources. The first would be contributed by albumin and transcuprein, which are immediately labeled with the radioactive tracer and rapidly deliver it to cells of the liver and mammary gland in lactation (Weiss and Linder 1985; Donley et al. 2002); the second would be from radiotracer in plasma Cp, which is incorporated within hepatocytes and appears in the blood later on, after tracer has virtually disappeared from the other two plasma proteins. (Recent evidence from cell culture studies confirms earlier observations from our laboratory; Campbell et al. 1981; Orena et al. 1986; Lee et al. 1993; Linder 2010; that Cp transfers copper directly to cells; Danny Ramos, David Mar, Michael Ishida, Maria Linder, unpubl. data.) In the current studies, we found that it took at least 2 h after radioactive copper injection for substantive amounts of labeled Cp to emerge in the blood.

While the mammary gland of the mice expressing the ATP7A construct retained less copper, both in terms of radioactive tracer and in terms of actual copper measured by atomic absorption, the livers of these mice had more tracer than in the WT. This is consistent with radiotracer effluxing back into the blood through ATP7A, emerging as ionic copper, and returning to the liver bound to albumin and transcuprein, the main organ taking up copper from these proteins (Fig. 6) (Lee et al. 1993; Linder 2010). The results of these studies clearly demonstrate for the first time the role of ATP7A of returning copper to the blood from a second cell type (the first being intestinal enterocytes), namely that of the mammary epithelium, and one where both copper “pumps” play important but probably very different specific roles.

Although initial analysis of the Tg hATP7A mice indicated expression of the latter in some hepatocytes (Ke et al. 2006), the current data indicate that this ectopic expression of ATP7A in hepatocytes did not significantly enhance rates of copper incorporation into Cp that entered the blood, nor did it increase metalation of this protein – as measured by ratios of holo to apoCp in the plasma, and by assays of Cp oxidase activity. Possibly, the fraction of hepatocytes‐expressing ATP7A in these mice was too low to cause any detectable alterations in Cp metalation coupled with the fact that the ATP7A may be primarily located on the plasma membrane (Ke et al. 2006) rather than the TGN where the metalation of Cp occurs.

Turning to our findings on the role of ATP7A in gestation, these showed that overexpression of ATP7A did increase the rate of copper transport to the fetus, suggesting that during much of gestation ATP7A is rate‐limiting for copper transport. The confounding result, however, was that when pups were sampled on day 1 after birth, the difference between the Tg and WT had disappeared. The total copper data for fetuses scattered from early through middle and even later levels of gestation strongly suggest that those with extra ATP7A initially have some advantage in gaining copper from their dams. Even if one pools the data from throughout pregnancy in Figure 4B, which show two parallel lines of increasing concentrations progressing with pregnancy stage, mean copper concentrations were slightly higher in the case of the Tg mice than in the WT. Moreover, concentrations in the placentae demonstrated the reverse phenomenon, consistent with excess ATP7A pumping more copper into the fetal circulation. This is good evidence that ATP7A‐mediated copper transport is the rate‐limiting step in the transfer of copper from mother to fetus during most of pregnancy.

However. by day 1 of lactation, after birth, the Tg pups no longer had more copper than the WT. One possible explanation is that at the time of sampling they had already used up the extra copper acquired during gestation for their growth and obtained less copper from the milk produced by the Tg dam, resulting in the change observed. Another possibility is that in the very last part of gestation, there was increased expression of placental mAtp7A in the WT, which allowed them to “catch up” with their transgenic counterparts. As calculated earlier, the oldest fetuses analyzed for total copper were about 85% of their birth weight, indicating they still had a few days in which to gain additional copper. Mammalian fetuses accumulate stores of iron, copper. and zinc in their livers before birth, which are used up during lactation (Linder 2010). Based on human studies, almost half of the excess iron stored in liver accumulates in the last 2 months of gestation. The same may be true for liver copper stores. As ATP7A is not associated with copper uptake by placental (or other) cells, but is expressed on the fetal side, this suggests that copper uptake is enhanced in the last part of gestation. Based on what is currently known, this would involve delivery of copper on plasma proteins to copper transporter 1 (CTR1) (Lee et al. 2002a; Lutsenko et al. 2007; Gupta and Lutsenko 2009; Wang et al. 2011) and/or to other copper transporters yet to be identified (Lee et al. 2002b; Kidane et al. 2012). In addition to albumin and transcuprein, Cp may be a donor, as we had previously demonstrated with i.v. injections of ^67^Cu‐labeled Cp into rats that showed a placental preference for copper from this protein (Lee et al. 1993). Moreover, Cp levels are increased in pregnancy (especially in the last third), making this copper more available. Our findings provide new insight into the mechanisms by which ATP7A participates in (and supports) the copper nutrition of the fetus, and how it may mitigate against the transfer of too much copper to the newborn after birth.

## Conflict of Interest

None declared.
